# Supplementary material for: Autonomous extraction of millimeter-scale deformation in InSAR time series using deep learning
Source: Nat Commun. 2021 Nov 10;12:6480. doi: 10.1038/s41467-021-26254-3 (PMC8581022; doi:10.1038/s41467-021-26254-3)
Supplement: Supplementary file 1 — Supplementary Information [file 41467_2021_26254_MOESM1_ESM.pdf]

Supplementary Materials for: Autonomous Extraction of Millimeter-scale Deformation in InSAR Time Series Using Deep Learning

Bertrand Rouet-Leduc[1,*], Romain Jolivet[2,3], Manon Dalaison[2], Paul A. Johnson[1], Claudia Hulbert[2]

[1]Los Alamos National Laboratory, Geophysics Group, Los Alamos, New Mexico, USA

[2] Laboratoire de Géologie, Département de Géosciences, École normale supérieure, PSL University, CNRS UMR 8538, Paris, France

[3]Institut Universitaire de France, 1 rue Descartes, 75005 Paris.

[*]To whom correspondence should be addressed; E-mail: bertrandrl@lanl.gov.

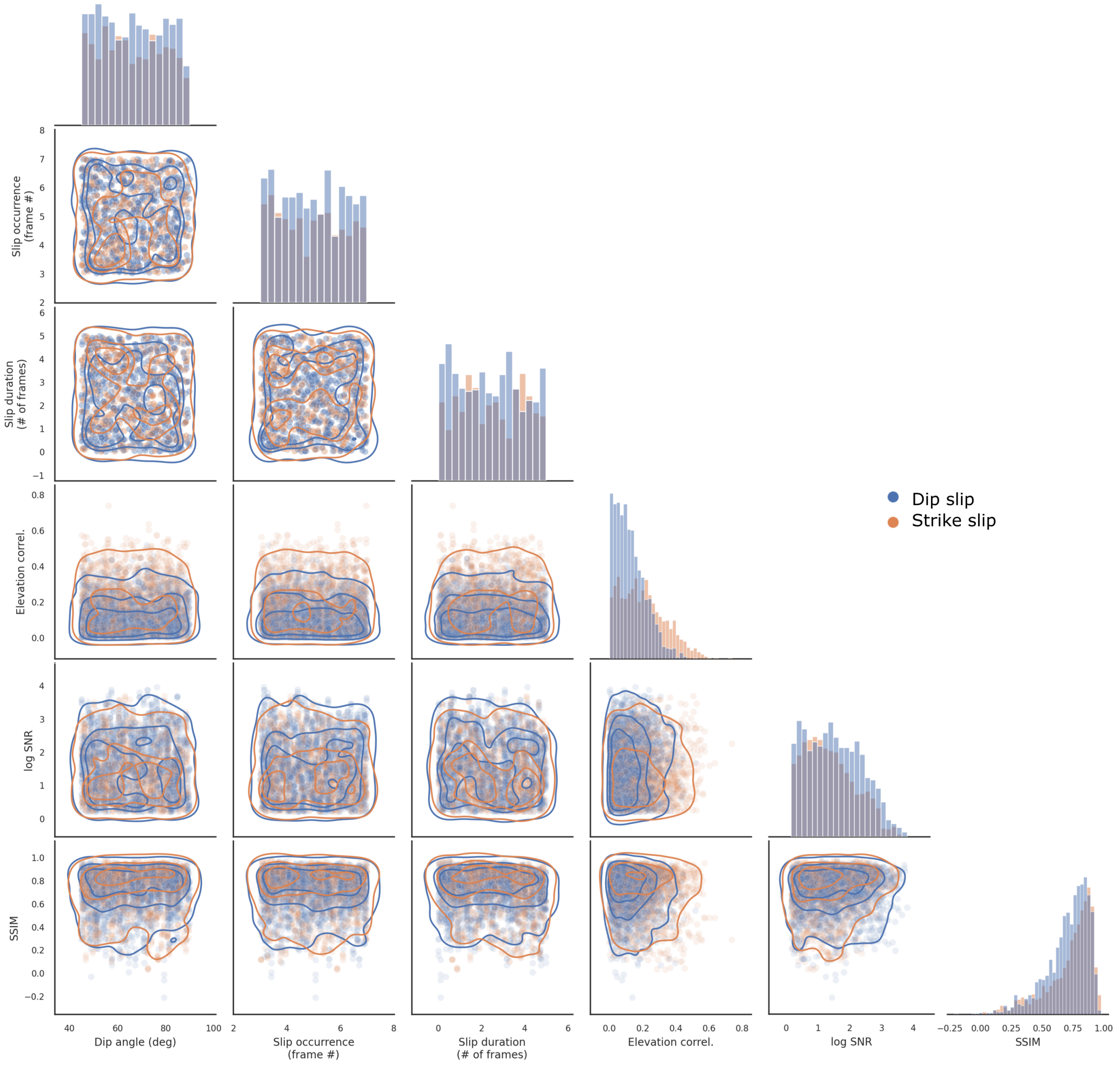

Figure S1: **Pair-wise distribution of the synthetic data and the performance of the model on fault deformation.** The off-diagonal plots show the pair-wise distribution of the data, as a function of signal to noise ratio (SNR), structural similarity index (SSIM, showing the performance of the model), slip duration (in number of frames), slip occurrence (timing of peak deformation in the input time series, in frame number), fault dip angle, and absolute normalized correlation between deformation and elevation, for strike slip (orange) and dip slip (blue). The diagonal plots show the distribution of each variable. Each dot is a synthetic time series, and the contours show kernel density estimates of the distribution, separately for dip slip and strike slip (in blue and orange, respectively). The first 4 plots of the bottom row show in particular little to no dependence of the performance of the model (SSIM) with respect to dip angle, slip occurrence and duration, slip type (dip-slip or strike-slip), or correlation (normalized) between deformation and elevation. Data at high SNR (SNR ≥ 1), where SSIM shows little dependence with SNR (see Fig. 2 of the main text). Test data, not seen by the model in training (5000 time series).

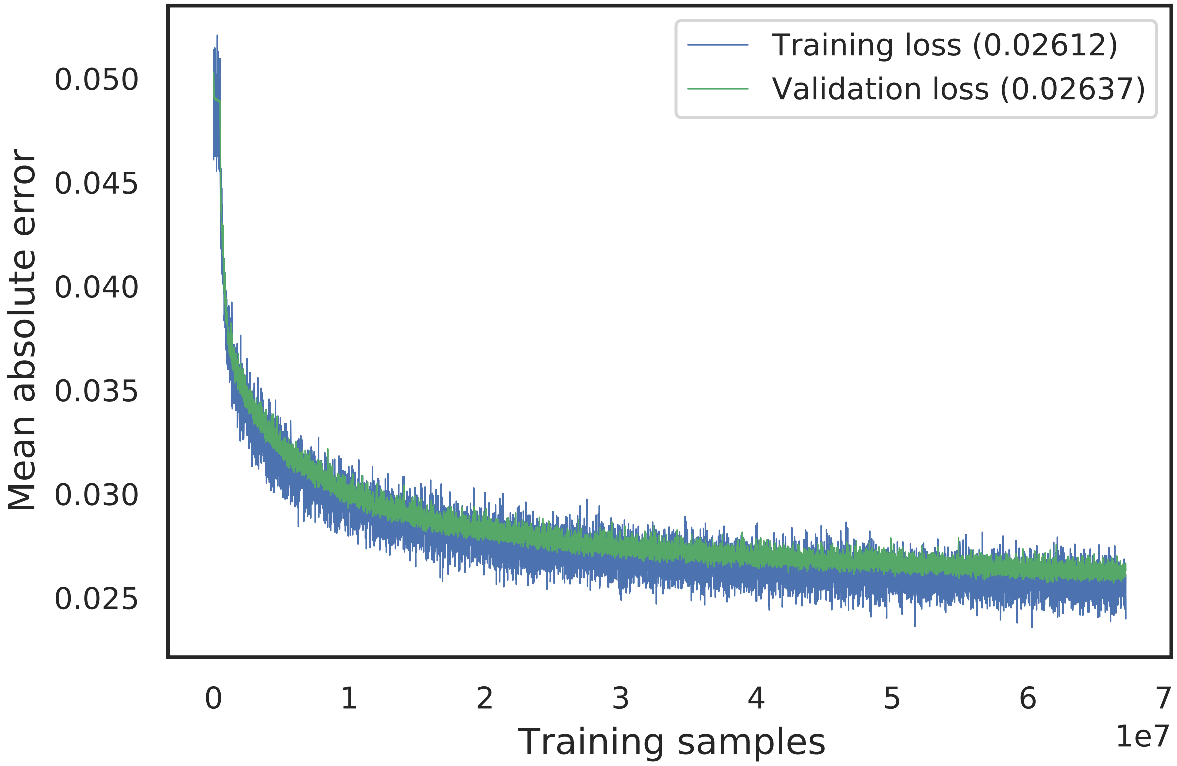

Figure S2: **Training curve of the deep denoiser.** Performance of the model in terms of mean absolute error (the loss of the model, that it tries to minimize in training), as a function of number of training sample it has seen. Blue: performance on batches of training data. Green: performance on validation data (1000 samples not used to adjust the parameters of the network, but only for evaluating how training progresses).

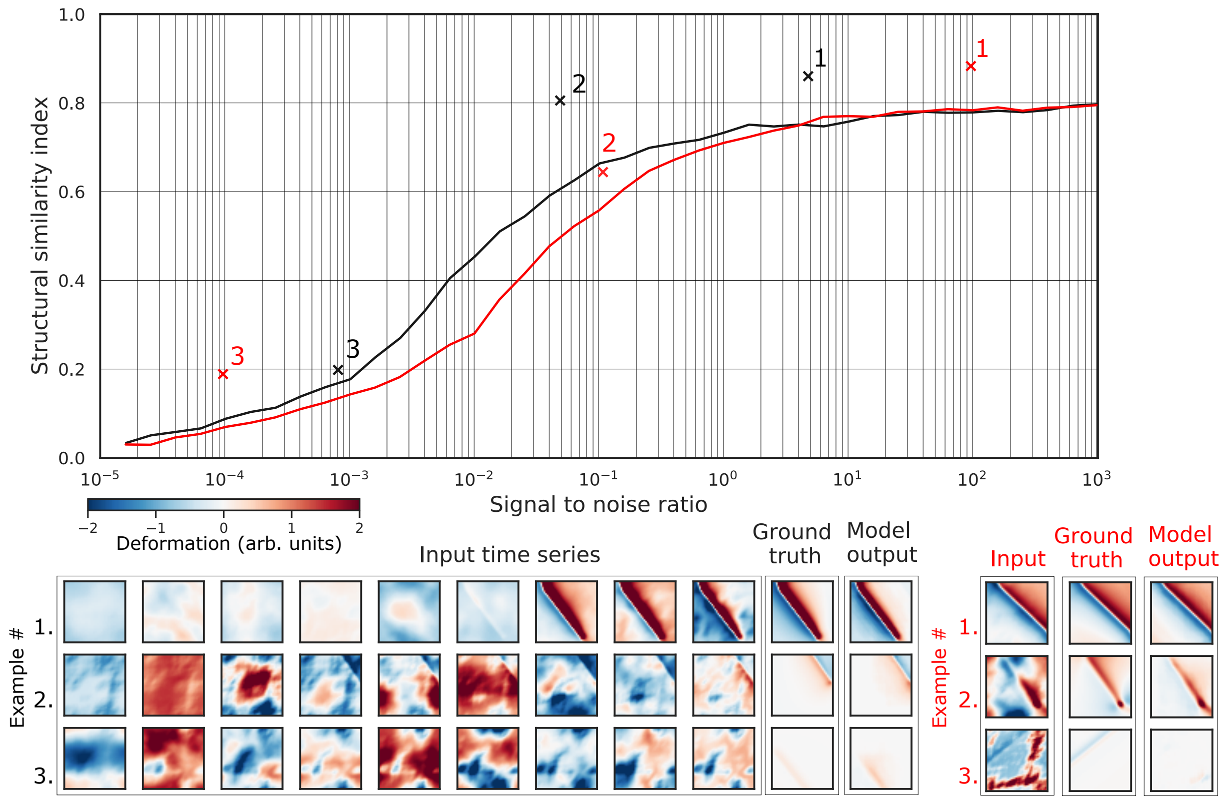

Figure S3: **Comparison with a deep auto-encoder trained on a single step.**. To evaluate the effect of the temporal filters learned by our model, we trained from scratch another model, trained to detect deformation in a single time step instead of 9. Everything is kept the same, as described in the main text. Top: Median performance (10^5 test samples) of the single step model (in red) and the 9-steps model analyzed in the main text and the rest of the supplementary (in black), as measured by structural similarity index (SSIM) between models output and deformation ground truth, as a function of signal to noise ratio (SNR). Bottom: examples of the data showing input, ground truth, its reconstruction by the deep autoencoders, 9-steps on the left and single step on the right. For a given threshold of 0.4 SSIM, the single time step model fails at 2% SNR and the initial 9-steps models fails at 0.6%, about a third the amount of noise. Note moreover that the 9-step model is very robust to false deformation (temporary fault-like noise), as shown in the supplementary figure Fig. S6, whereas the single-step model has no way to make the distinction.

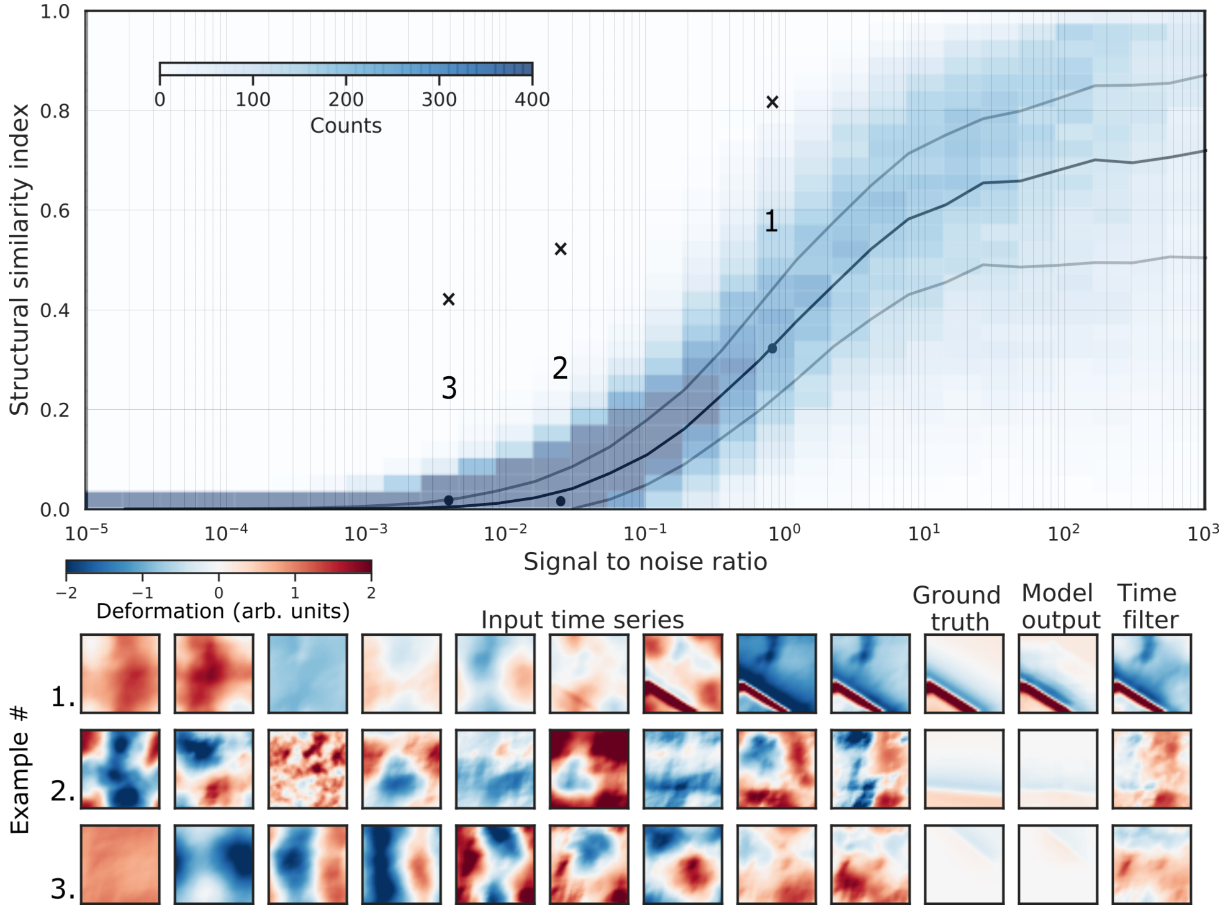

Figure S4: **Comparison with a simple temporal filter.** The temporal filter evaluated is the average of the time series after the deformation on the fault starts minus the average of the input time series before the deformation on the fault starts. This filter is evaluated exactly as in Fig. 2 of the main text. Top: Performance of the filter, as measured by structural similarity index (SSIM) between filter output and deformation ground truth, as a function of signal to noise ratio (SNR). Shades of blue show the distribution of SSIM as a function of SNR (counts per bins for 10^5 test samples). The black and gray lines show the median and 25th and 75th percentile of the SNR bins, respectively. Bottom: examples of the data showing input time series, ground truth, its reconstruction by the deep autoencoder, and its reconstruction by the simple temporal filtering, for different signal to noise ratios. The matching numbers correspond to the temporal filter for the crosses in the plot above, and to the autoencoder for the dots.

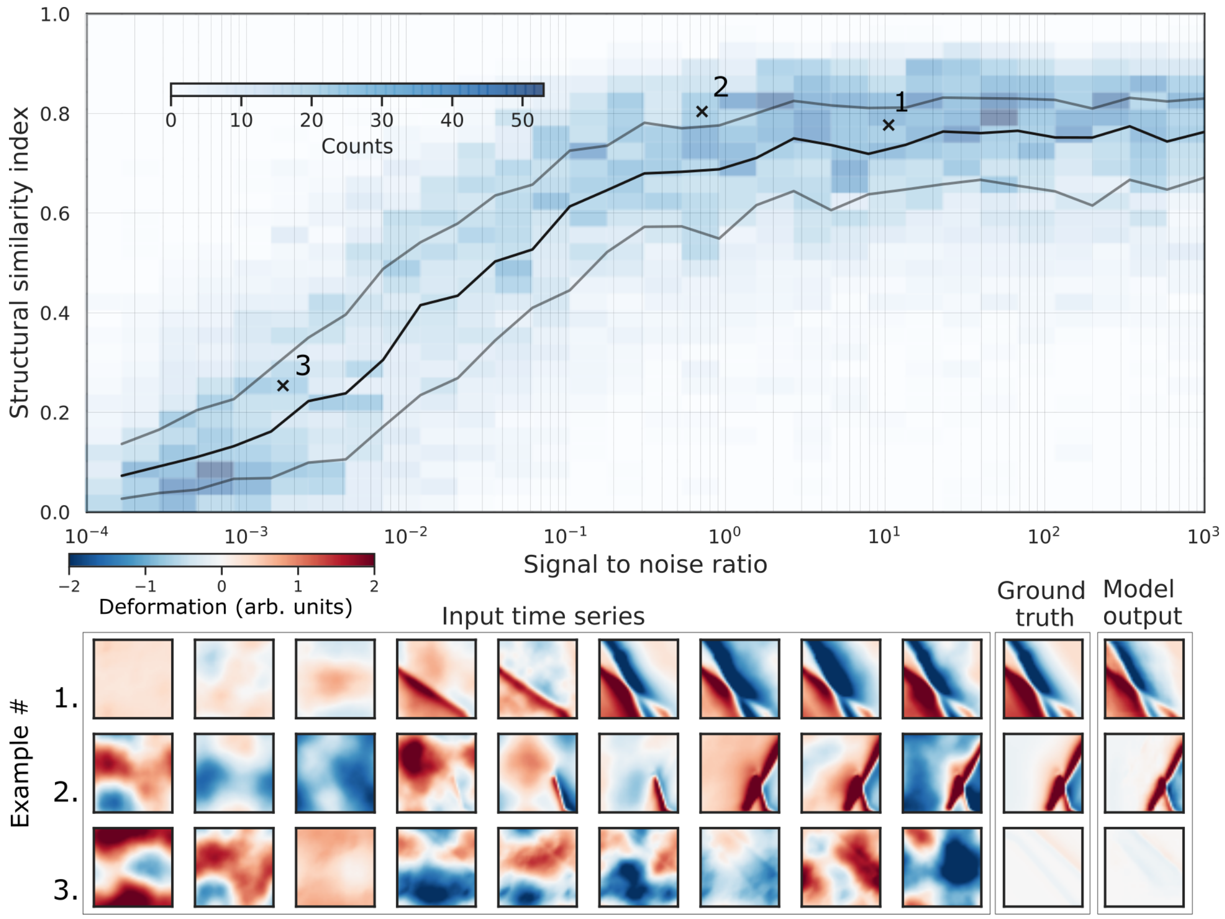

Figure S5: **Performance on synthetic test data: slip on multiple faults.** Top: Performance of the reconstruction of deformation caused by independent fault patches slipping, on synthetic noisy time series, as measured by structural similarity index (SSIM) between model output and deformation ground truth, as a function of signal to noise ratio (SNR). Shades of blue show the distribution of SSIM as a function of SNR (counts per bins for 10^4 test samples). The black and gray lines show the median and 25th and 75th percentile of the SNR bins, respectively. Bottom: examples of the data showing input time series, ground truth, and its reconstruction, for different signal to noise ratios, shown with matching number in the plot above. Note that the model has only been trained on single patches and yet performs almost as well for reconstructing the deformation caused by slip on multiple fault patches.

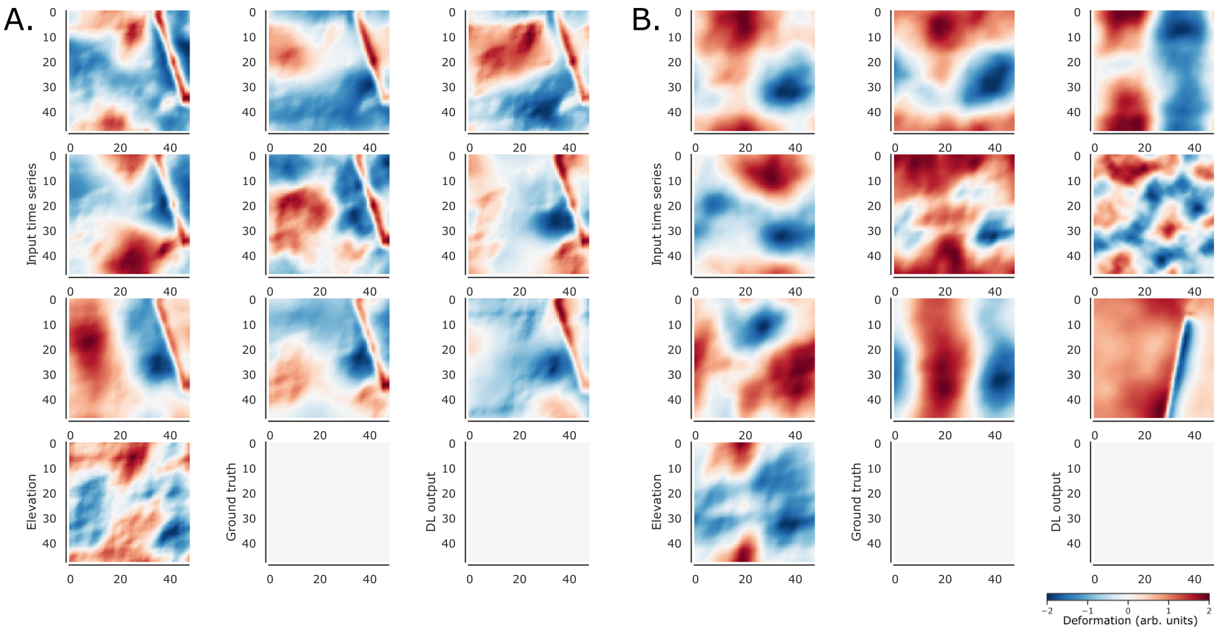

Figure S6: **Deformation in the first and last time elements of the input time series.** The deep auto-encoder uses the first and last time steps of the input time series as references. As shown in the examples above, it does not reconstruct deformation that was already there (present in the first time step, as in A) or starts at the end of the series (appears in the last time step, as in B).

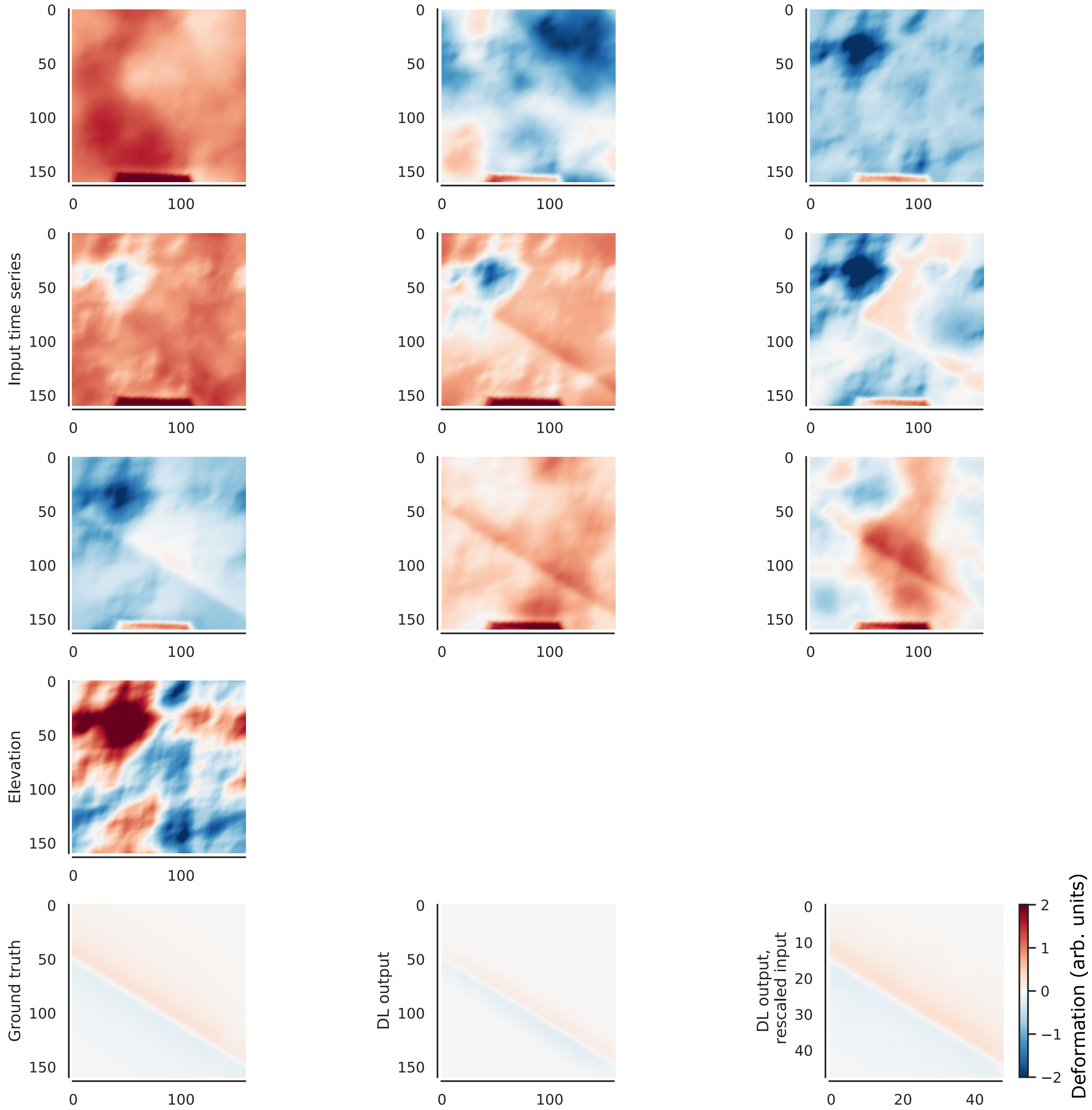

Figure S7: **Effect of resolution and maximum wavelength** The deep auto-encoder is trained on small synthetic time series (48 × 48 pixels), which limits the deformation wavelength it can resolve. As shown here, deformation beyond 20 to 30 pixels can be missed by the model, but down-sampling the input before applying the model enables to capture the full deformation. As in the previous figure, pre-existing deformation is not reconstructed by the model.

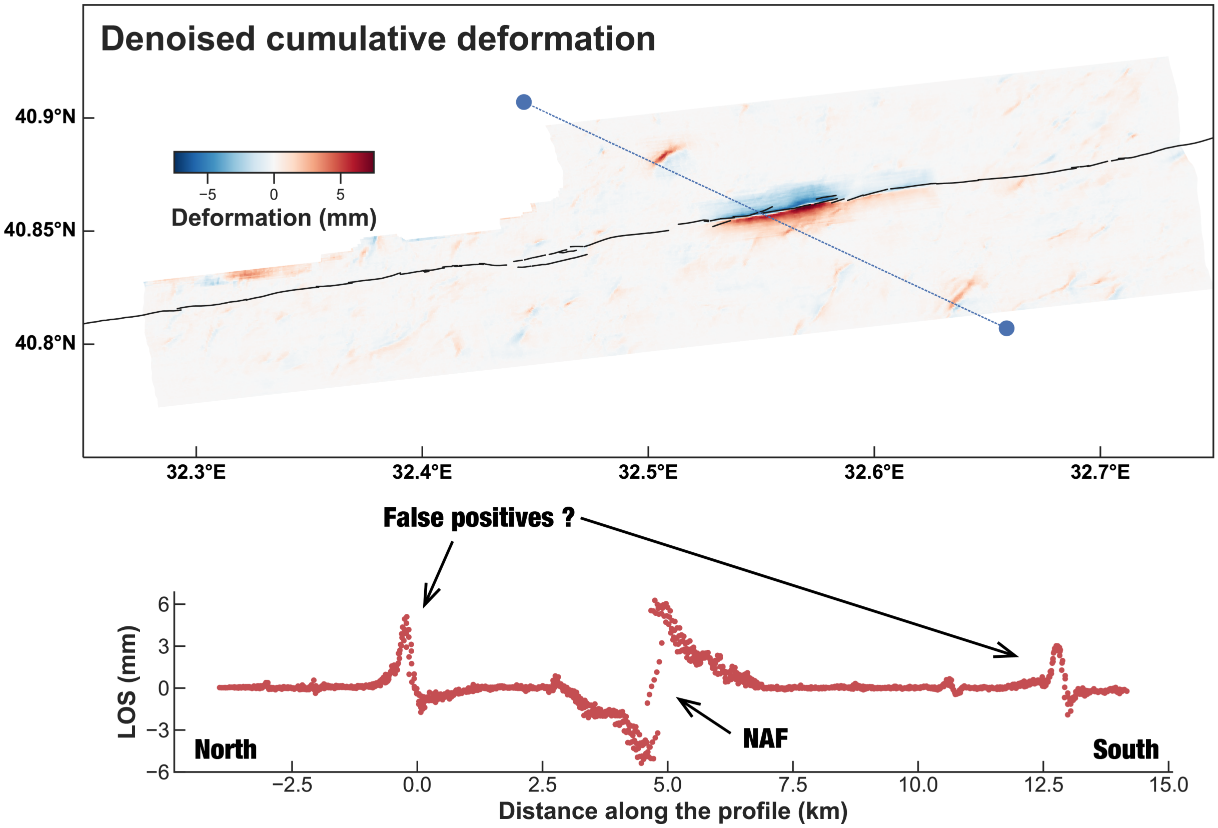

Figure S8: **Likely false positives off the North Anatolian Fault.** Top: output of the deep denoiser applied to COSMO-SkyMed data over the North Anatolian Fault (Same as in Fig. 3 of the main text). Bottom: cross-section (above in blue) across the fault and across two sets of signals off the fault, that we interpret as remaining noise.

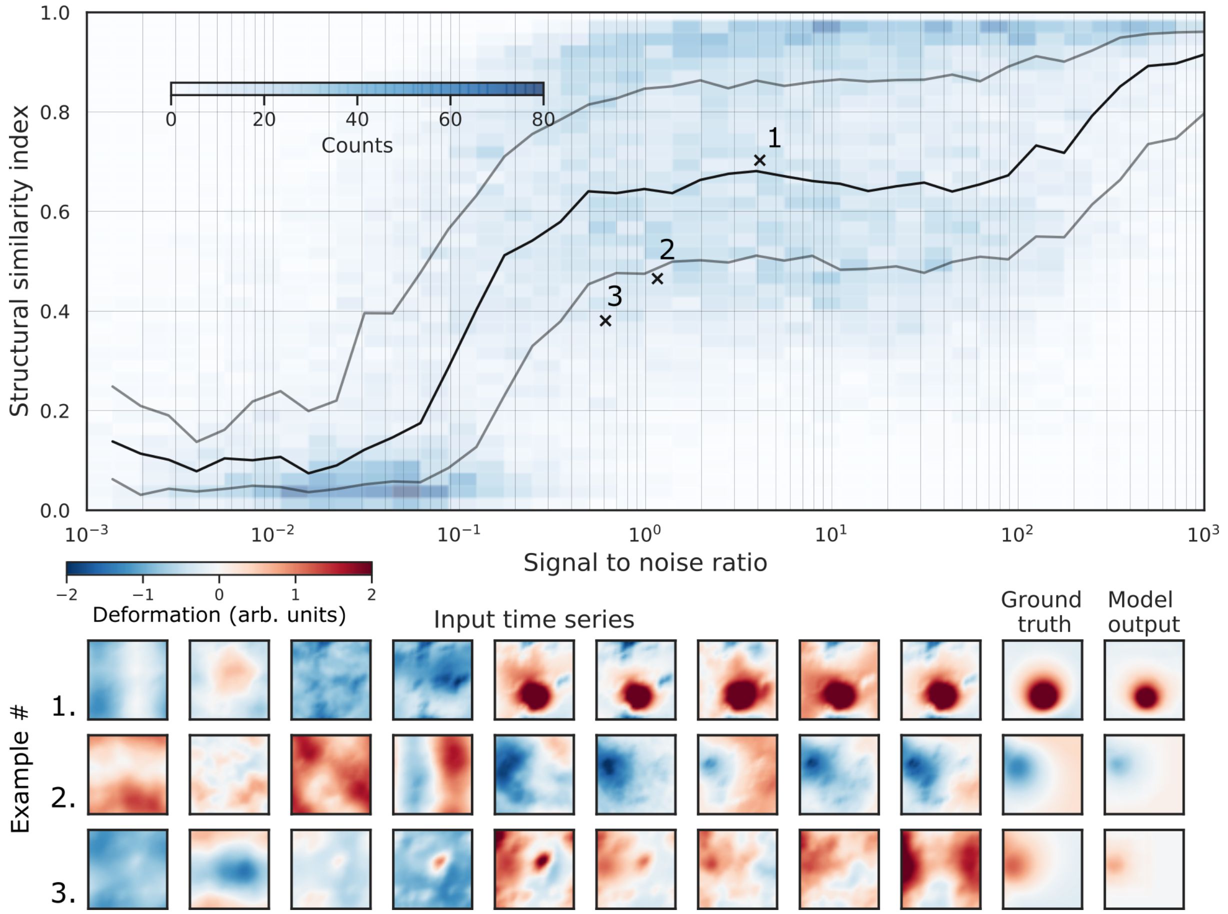

Figure S9: **Performance on synthetic test data: pressure sources.** Top: Performance of the reconstruction of deformation caused by pressure sources by our deep auto-encoder, on synthetic noisy time series, as measured by structural similarity index (SSIM) between model output and deformation ground truth, as a function of signal to noise ratio (SNR). Shades of blue show the distribution of SSIM as a function of SNR (counts per bins for 10^4 test samples). The black and gray lines show the median and 25th and 75th percentile of the SNR bins, respectively. Bottom: examples of the data showing input time series, ground truth, and its reconstruction, for different signal to noise ratios, shown with matching number in the plot above. The model can recover with reasonable fidelity (SSIM> 0.4) deformation signals with SNRs down to 20%, compared with 0.5% for deformation on faults. Pressure sources of deformation are also arguably harder to distinguish with the eye than deformation on faults.

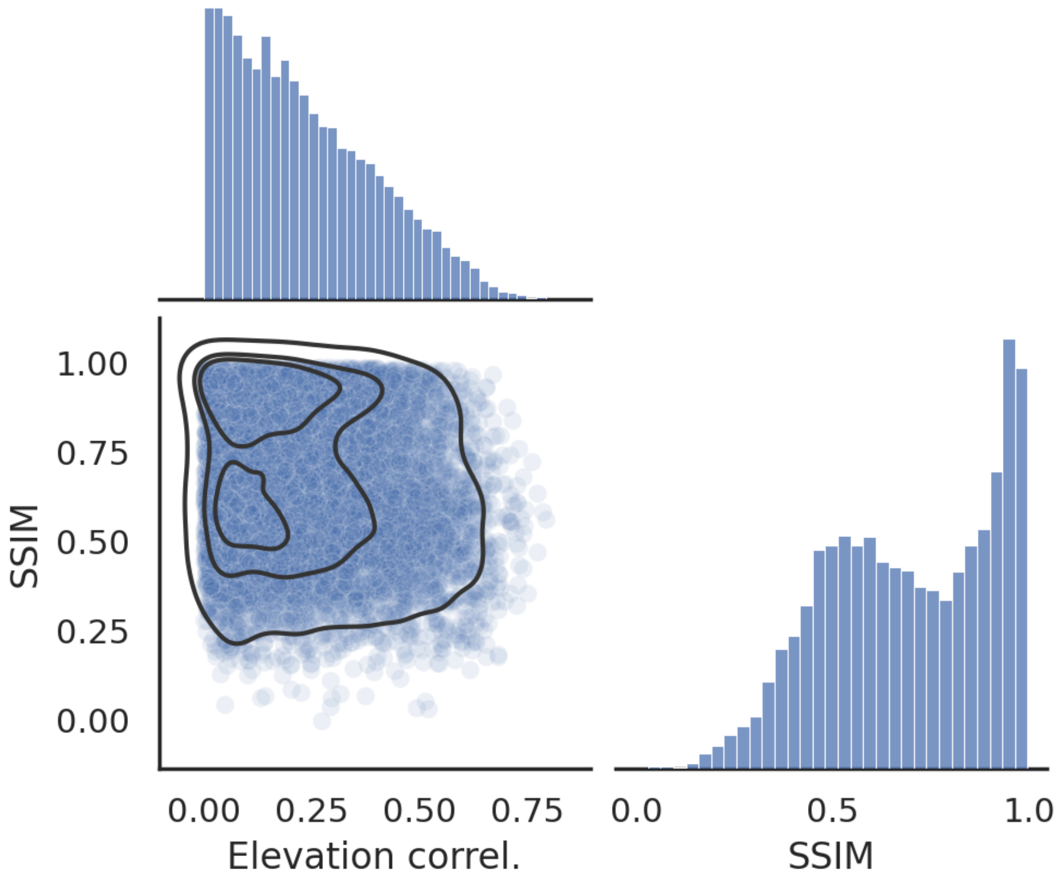

Figure S10: **Distribution of the performance of the model for detecting pressure sources, as a function of correlation between topography and deformation.** Test data, not seen by the model in training (10000 time series, same data as figure above). We observe very little effect of the correlation between signal and elevation on the performance of our model.

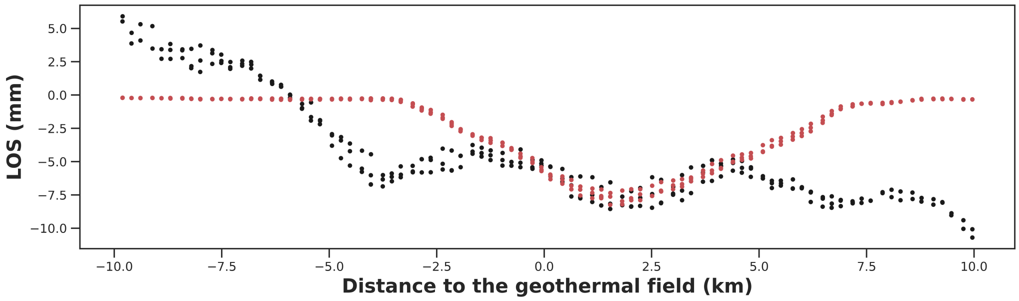

Figure S11: **Application to real data: the Coso Geothermal Field in California.** Application of the deep denoiser to Sentinel 1A-B time series from 2016-04-14 to 2016-11-16, that spans the Coso Geothermal Field in California. Cross sections as indicated in Fig. 5 in the main text. Black: cross section across the cumulative displacement in the input time series. Red: cross section across the denoised output of the model.

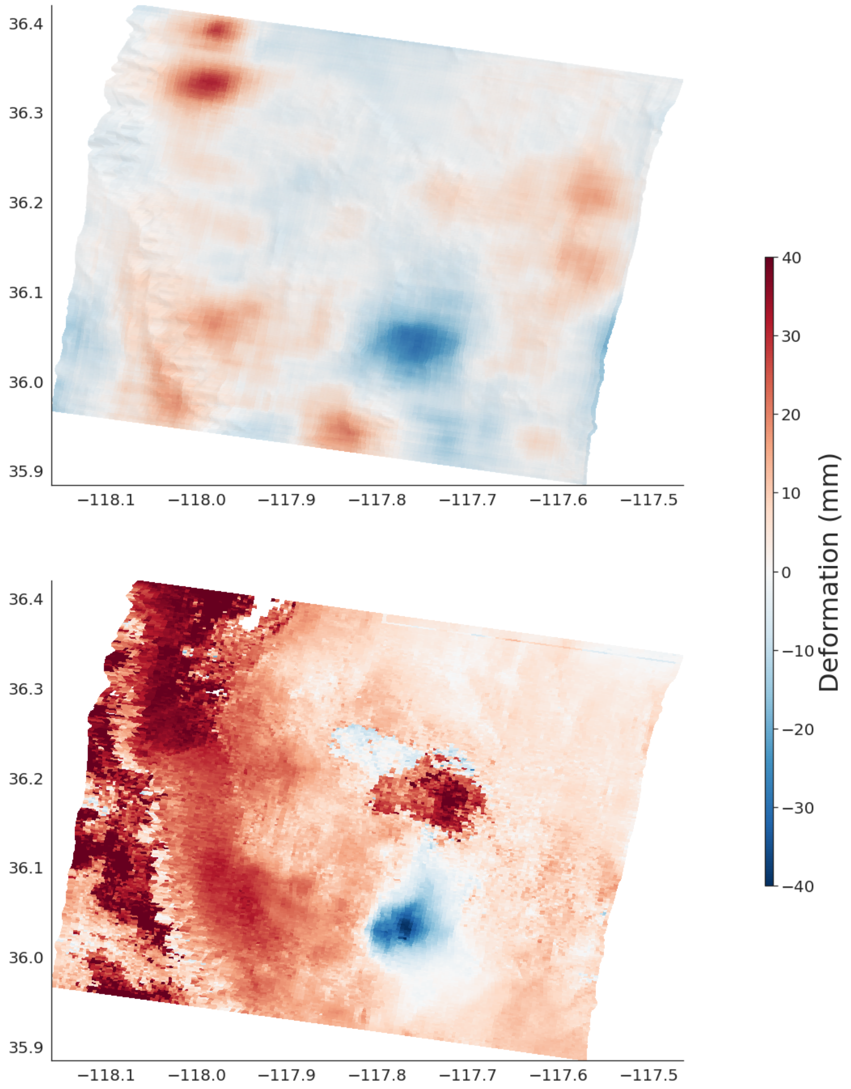

Figure S12: **Application to real data: the Coso Geothermal Field in California.** Top: cumulated deformation from all the transient deformation detected by our deep denoiser. Bottom: cumulated deformation at Coso from 2015-12-16 to 2019-05-29 obtained from Sentinel 1A-B. Both deformation fields are strongly correlated, showing that most of the deformation at Coso takes place as transient deformation (which is what the model is sensitive to).

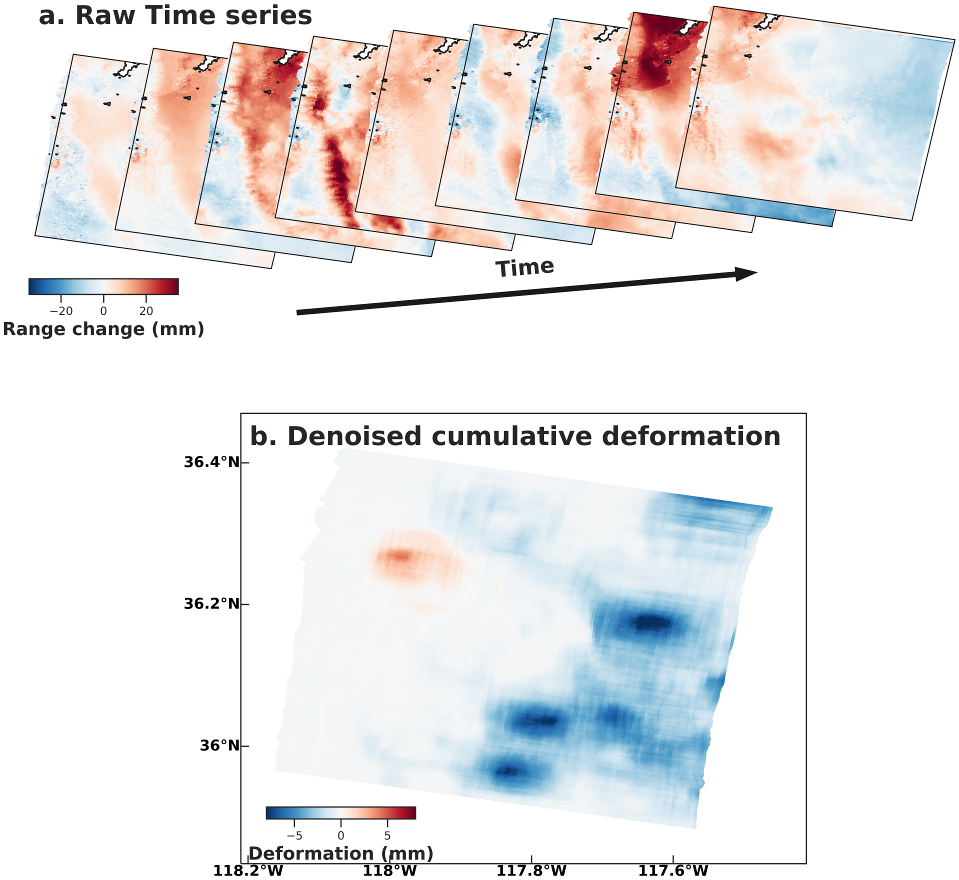

Figure S13: **Application to real data: the Coso Geothermal Field in California.** As in Fig. 5 in the main text, the deep denoiser is applied to the time series obtained from Sentinel 1A-B, but for a different set of images, from 2015-12-16 to 2016-07-19. **a.** Input raw time series of 9 successive images from Sentinel 1 data. Color is the apparent range change between the satellite and the ground along the LOS. **b.** Denoised cumulative deformation as output by our deep auto-encoder. Color is ground deformation in the LOS.

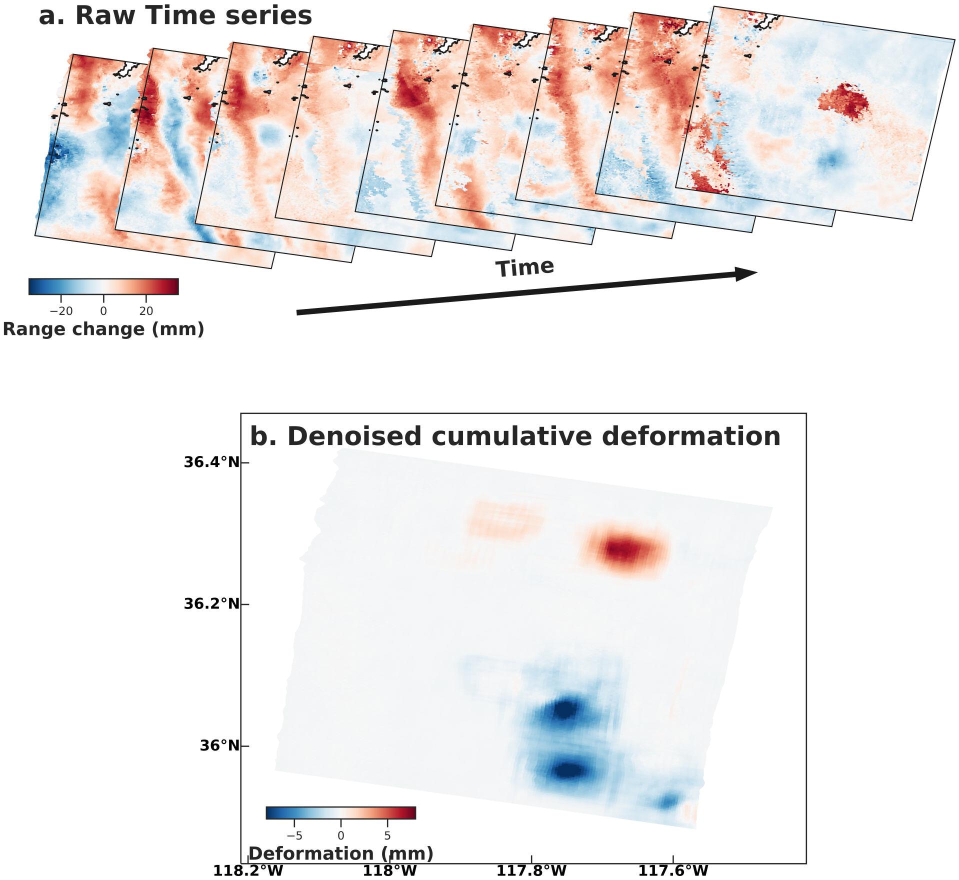

Figure S14: **Application to real data: the Coso Geothermal Field in California.** As in Fig. 5 in the main text and Fig. 4 of the Supplementary, but for yet another set of images, from 2016-09-29 to 2017-02-20. **a.** Input raw time series of 9 successive images from Sentinel 1 data. Color is the apparent range change between the satellite and the ground along the LOS. **b.** Denoised cumulative deformation as output by our deep auto-encoder. Color is ground deformation in the LOS.

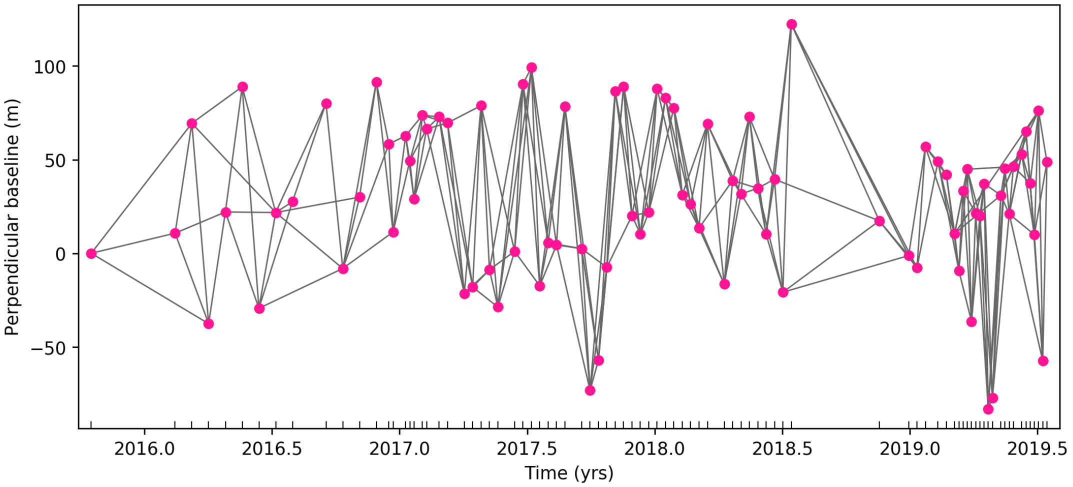

Figure S15: **Small baseline interferometric network for the Sentinel 1 data.**
